# The Case for Understanding Interdisciplinary Relationships in Health Care

**DOI:** 10.31486/toj.22.0111

**Published:** 2023

**Authors:** Jasmine L. Warren, Jimmie S. Warren

**Affiliations:** ^1^ Clinical Research, Ochsner Medical Center–Kenner, Kenner, LA; ^2^ Department of Business Administration and Accounting, Mississippi Valley State University, Itta Bena, MS

## INTRODUCTION

An interdisciplinary relationship, defined as 2 or more disciplines collaborating to accomplish a common goal, is considered a general and broad form of collaboration.^1^ For example, an automobile company and a computer software company can work in partnership to offer specific automotive features such as navigation systems, sensors, and internet radio that can create a competitive advantage in the market. The concept of interdisciplinary relationships is highly valued throughout many business and academic areas. Interdisciplinary relationships can ensure that customers’ needs are met, the cost of operations decreases, and customer loyalty is not only sustained but can also increase and perhaps attract new customers.^2^ In academia, instructors can engage students in broad learning opportunities by overlapping portions of their curricula. Colton and Surasinghe conducted an observational study of a collaboration between the English and biology disciplines at Clemson University.^3^ Biological science majors were required to take an English course that emphasized scientific writing to give students the opportunity to learn about thinking critically and communicating ideas clearly across different fields of study.^3^

These types of interdisciplinary strategies can be applied in the health care industry. Interest has increased in the concept and the efficacy of interdisciplinary collaborations in health care areas such as research hospitals, allied health, and primary care.^4^ Discussions have resulted in attempts to identify ways to implement interdisciplinary collaborations.^5^ However, personnel in some areas of health care do not understand the value of training to improve interdisciplinary collaborations.^6^ With the proper training of personnel, such interdisciplinary collaborations can be established and can potentially result in better patient care, reduced costs, expedited delivery of patient information, and an increase in perceived patient quality of care.^5^

## INTERDISCIPLINARY RELATIONSHIPS

Health care collaborations, defined as “an interpersonal process characterized by health care professionals from multiple disciplines with shared objectives, decision-making together to solve patient care problems,” are considered a specific kind of interdisciplinary relationship.^5^ The most common health care collaboration is between physicians and nurses. These medical professionals work together on a daily basis to care for patients in clinics, hospitals, and home health care environments. Physicians and nurses understand that their work practices are complementary and impact patient outcomes. Establishing a professional environment that promotes communication, respect for each discipline, and high morale can lead to optimal health care outcomes for patients.^7^

Kerrissey et al explored the relationship between structural integration characteristics of several health care organizations and how integrated care was perceived by patients.^8^ The structural integration characteristics, taken from the National Study of Physician Organizations, included organizational size, interdisciplinary mix, technologic capacity, and the process for managed care. Patient perceptions of integrated care were assessed across 6 areas that included specialist, provider, and staff knowledge about the patient; provider support; and test result communication. Based on the responses to a validated national survey, the study showed that patients responded favorably regarding communication of test results and unfavorably regarding medication and home health management support. In addition, the study showed that patients had a favorable perception of health care staff knowledge about their medical history.^8^

Tremblay et al looked at the association of interdisciplinary teamwork intensity (high vs low) with patient-reported experience measures (PREMs) for outpatients undergoing cancer care.^4^ The PREMs included prompt access to care, person-centered response, quality of patient-professional communication, and continuity of care. Outpatients being treated for cancer at health care facilities defined as having high intensity interdisciplinary teamwork had more highly favorable perceptions of the 4 PREMs than outpatients being treated at health care facilities with low intensity interdisciplinary teamwork.

Examples of interdisciplinary collaborations are health centers that provide both primary care and dental care and centers that provide primary care and mental health care.^9^ Such health centers must be able to use electronic medical record systems in an integrative manner so that all of a patient's records—both medical and dental, for example—are easily accessible. The integrated records can improve patient care and reduce costs.^9^ However, Hilton explained that the trial run of a collaboration between primary care and dental care revealed flaws such as lack of proper and specific training for collaborative health care teams, lack of proper flow of patient information between health care specialists, and lack of enthusiasm to seek diverse perspectives to diagnose and interpret patient data and information.^9^ More data should be collected on these challenges to address the problems and establish the efficacy of interdisciplinary collaboration at such collaborative health centers.^8,9^

Another example of interdisciplinary collaboration is accountable care organizations that mandate interdisciplinary relationships in the form of teams consisting of a primary care physician, nursing staff, and specialists who care for patients.^10,11^ This kind of health care collaboration has been suggested as an effective option for lowering the cost of patient care and improving patient outcomes.^10^ Kaufman et al reviewed 42 articles that assessed the effect of accountable care organizations on health care utilization, processes of care, and outcomes.^12^ The articles included in the review were 24 Medicare studies, 5 Medicaid studies, and 13 private payer studies. The review showed consistent relationships with accountable care organization implementation and outcomes for all providers in terms of a decline of inpatient use, a decline in emergency room visits, and improved quality measures for preventive care and chronic disease management.^12^ Seven of the articles that assessed patient experiences or clinical outcomes showed that patient health care was not worsened by accountable care organizations.

Primary care physicians have the leadership position in the team collaboration because they are traditionally patients’ first point of contact when seeking care.^9^ A 2005 report published in the *Journal of Interprofessional Care* showcased how the University of Hawaii at Manoa John A. Burns School of Medicine offered community-based interdisciplinary training for medical students.^13^ The program was designed to train and to produce physicians who would elect to work in primary care disciplines, practice in underserved communities, and work in an interdisciplinary environment.^13^ Other health care–based teaching facilities and universities have implemented similar courses on interdisciplinary relationships, including George Washington University, the University of Missouri School of Medicine, Louisiana State University Health Sciences Center, and the University of Medicine and Dentistry of New Jersey. These academic institutions have offered training programs with a focus on strengthening medical students’ knowledge and skills in preparation to work in interdisciplinary medical environments. These academic community–based medical trainings were also intended to provide continuing medical education opportunities.^14^

## CONFLICT RESOLUTION

Health care collaboration efforts can be difficult, with unique challenges such as the need for funding, specific training for collaborative teams, appropriate flow of patient information among specialists, and enthusiasm for seeking diverse perspectives to diagnose and interpret patient information. Lack of funding and resources can hinder collaboration.^12^ When resources are stretched to meet the demand for services, investments needed to establish interdisciplinary teams may be reduced. For example, organizations that offer palliative care are aware of the benefits of interdisciplinary teams; however, resources are scarce because of the rising cost of care, low reimbursement rates, and increased patient populations.^15^ Hodiamont et al suggested the use of the complex adaptive systems theory to address the “reciprocal, nonlinear relations and uncertainties” of palliative care.^16^ The study aim was to describe attributes that contribute to the complex issues associated with palliative care from the perspective of the professional caregiver and to establish a framework for identifying problems and solutions. The allocation of additional funding to invest in integrative health care technology (ie, medical records, real-time health vitals documentation, and patient portal documentation) could potentially address some of the interdisciplinary relationship challenges of palliative care. An example is the ability to share patient records between a primary care physician and endocrinologist who are both providing medical care to the patient.^17^

Another challenge to interdisciplinary collaboration efforts is the need to develop standardized policies and training that incorporate the knowledge and skill sets of multiple health care providers.^18^ In hospitals, conflicts can arise between the medical staff and the cleaning staff about the cleaning and sterilization methods used for patient rooms. This conflict has become an area of concern because of the increased reports of hospital-acquired infections.^19^ Differences in medical background training can present challenges to working together for the benefit of the patient when multiple departments are involved. Petri suggests educating individuals to be open in communication and to respect each health care professional's discipline.^5^ Health care administrators can champion and promote these solutions through organizational workshops and policies that promote collaboration. Van Houtven et al suggested the use of the complexity science theory (the study of complex systems) in conjunction with an adapted form of an intervention called Connect.^20^ The Connect intervention was created for the Department of Veterans Affairs to train nursing home personnel on improving connections with colleagues, improving the flow of information, and improving cognitive diversity to reduce patient falls. Training methods included telling stories and role playing, relationship mapping, mentoring, and monitoring communication patterns and use of strategic interaction methods. Colón-Emeric et al showed that the Connect intervention was successfully used to improve communication and organizational safety.^21^ Team members (staff) reported significant increases in their ability to recognize and identify weaknesses in communication, as well as making a more concerted effort to seek varying perspectives on understanding and acting on patient information. Staff also reported the creation of better and more effective care plans, a heightened respectful environment, and an improvement in relationships with colleagues.^21^

Measures focused on addressing challenges to interdisciplinary relationships point to a potential shift in the cultural atmosphere in the health care industry toward supporting and promulgating positive communication and synergistic collaborative efforts. This cultural shift can aid in mitigating the negative impact on health care outcomes resulting from poor relationships among health care professionals.^22^ Kock et al assessed the impact of implementing interprofessional practice (IPP) on the perceptions, attitudes, and understanding of health care professionals.^6^ IPP was being implemented as a way to address interdisciplinary relationship challenges in health care practice. The findings of the study suggested that health care professionals lacked the understanding of and the ability to practically apply IPP. In addition, health care processes were perceived as hindrances to the implementation of such interdisciplinary practices. The study also showed that health care professionals expressed the necessity for interprofessional relationships, an environment that fosters IPP, and an environment that fosters a level of communication for changing the current health care work practice.^6^

## CONCLUSION

Improved patient care and safety are the goals of establishing effective interdisciplinary relationships among the members of health care teams. The most common interdisciplinary relationship in health care is the one between physicians and nurses. Historically, collaborations among medical professionals of the same discipline have received more attention than interdisciplinary collaborations. However, the lack of attention to interdisciplinary collaborations is being addressed via efforts to educate health care personnel and to reduce costs by promoting collaborative processes. Health care providers must be willing to have open communication with one another. In addition, adjustments to medical information systems to better communicate patient information, creation of health care education curricula to include multidisciplinary collaboration training, changes to health care policies, and structural changes to health care facilities are efforts suggested in the literature to accommodate the implementation of interdisciplinary relationships. Additional research is needed in the areas of communication, structural changes to medical facilities and medical processes, and the curricula to better understand and effectively promote the efficacy of implementing interdisciplinary relationships in health care.
